# Research progress on the synthesis process, detection method, pharmacological effects and application of glycocholic acid

**DOI:** 10.3389/fphar.2024.1492070

**Published:** 2025-01-03

**Authors:** Jiahui Li, Chungang Zhang, Yang Wang, Minyuan Tian, Chao Xie, Heng Hu

**Affiliations:** ^1^ College of Pharmacy, Liaoning University of Traditional Chinese Medicine, Dalian, China; ^2^ Department of Pharmacy, Changzhi Medical College, Changzhi, China; ^3^ Qimeng Co., Ltd., Chifeng, China

**Keywords:** glycocholic acid, synthetic process, pharmacological effect, absorption enhancer, research progress

## Abstract

**Objective:**

This review aims to summarize the research progress of glycocholic acid to promote its broader development and application.

**Methods:**

This article collects relevant literature from databases such as Science Direct, PubMed, Web of Science, Google Scholar and CNKI from the establishment to 2024, systematically organizing and analyzing aspects of glycocholic acid including its physicochemical properties, synthesis and extraction techniques, detection methods, pharmacological effects, mechanisms of action, clinical research, and application as an excipient.

**Results:**

Glycocholic acid, as a key conjugated component in bile acids, exhibits various pharmacological effects such as anti-inflammatory and antioxidant activities. Nevertheless, current research on glycocholic acid is insufficient, with synthesis techniques requiring improvement, limited application of detection technologies, and a need for in-depth exploration of its pharmacological mechanisms. Due to its amphiphilic molecular structure, glycocholic acid is primarily used as a pharmaceutical excipient.

**Conclusion:**

This review summarizes the existing research on glycocholic acid, indicating that future research should strengthen work in this field, including improving synthesis processes and enhancing the sensitivity of detection technologies, to provide a scientific basis for the development of new formulations and drug combinations, thereby promoting the advancement of traditional Chinese medicine.

## 1 Introduction

With a history of more than 1,000 years in China, Chinese medicine is primarily derived from natural animals and plants and has been widely used for its proven efficacy, low toxicity and other benefits. Bile, an essential component of animal medicines produced in the digestive fluid of the liver, has a long history of use in traditional Chinese medicine (Wang and Shi, 2000). In addition, in some regions, people use bile in specific food therapy or culinary practices, such as cattle bile powder, which is a dried product of bile, appearing as green-brown or brownish chunks or powder. It has been proven that taking it alone with boiled water or in combination with fleeceflower root, poria cocos, or sophora twig can have effect such as clearing heat, detoxifying, promoting bile secretion, anti-inflammatory, pain relief, and relieving constipation. The Shennong Bencao Jing from the Han Dynasty records the use of carp bile to treat redness, swelling and pain in the eyes, blindness, deafness impotence, etc., while Zhang Zhongjing in the Jin Yi Lun documents the functions of pig bile, such as suppressing coughs and relieving asthma symptoms, as well as its anti-inflammatory and anti-bacterial properties; Li Shizhen included more than 30 kinds of bile acid-containing herbs such as bull bile, sheep bile, bear bile, pig bile, etc., in his Bencao Gangmu, all of which are listed in the Dictionary of Chinese Medicine because of their strong medicinal effects, significant curative effects, and abundant resources, making them widely used in pill powder or other traditional Chinese medicine or Western medicine preparations (Zhao and Li, 2005).

**FIGURE 1:**
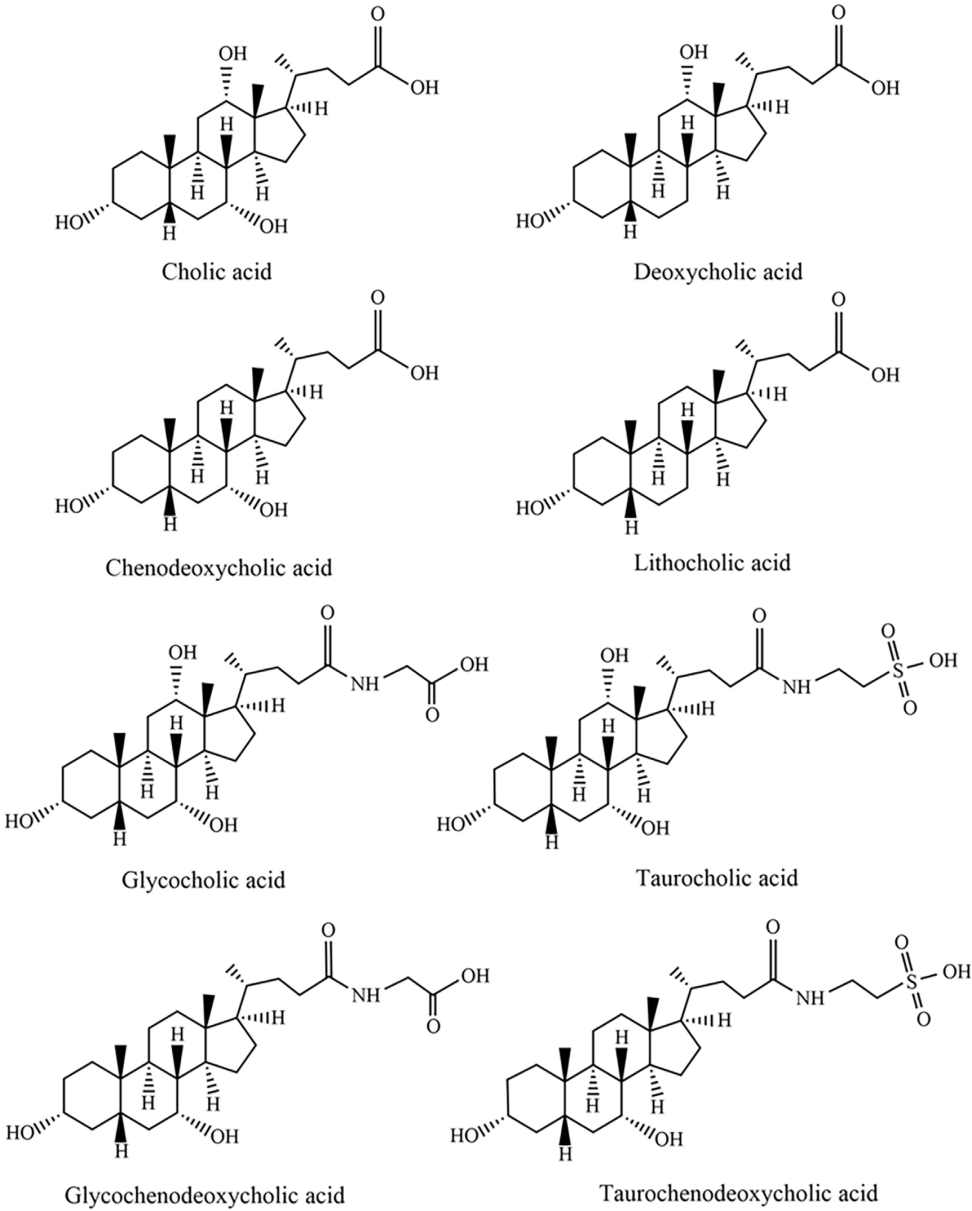
The structure of each bile acid.

Bile acids and bile salts are the primary bioactive components of animal bile that exert therapeutic effects. Bile acids are steroidal amphipathic molecules (Russell, 2009), which can be categorized into free bile acids and conjugated bile acids. The former includes cholic acid (CA), dexycholic acid (DCA), chenodeoxycholic acid (CDCA), and lithocholic acid (LCA), while the latter mainly consists of glycocholic acid (GCA), taurocholic acid (TCA), glycochenodeoxycholic acid (GCDCA), and taurochenodeoxycholic acid (TCDCA). The specific chemical structures of each component are illustrated in Figure 1.

Glycocholic acid, a bile acid complex synthesized in the liver from bile acids and glycine, is abundant in the bile of various animals. It plays a crucial role in enhancing lipase activity, catalyzing fat breakdown, and promoting bile secretion (Feng et al., 2014). Recent studies have revealed its additional therapeutic properties including anti-inflammatory, antipyretic, antioxidant, and antibacterial effects (Huang, 2019). Under normal physiological conditions, the liver absorbs glycocholic acid from the bloodstream, where its concentration remains low. However, when liver cells are damaged or impaired in their ability to excrete bile acids, peripheral blood levels of glycocholic acid is elevated. Consequently, glycocholic acid may serve as an early diagnostic indicator of abnormalities related to liver and bile function (Li et al., 2010; Zhao et al., 2019). Glycocholic acid as a raw pharmaceutical ingredient is widely used in the medical field, and the demand for it in clinical practice in our country is also constantly increasing. However, there are few reports on the research progress of glycocholic acid both domestically and internationally. Therefore, this article focuses on glycocholic acid and summarizes its structural properties, synthesis and extraction, detection methods, pharmacological effects, clinical research, significance of testing, and applications as an excipient. This aims to fill the gaps in the related field and provide references for guiding clinical medication.

## 2 Methodology for literature search

A literature search was conducted on databases including Science Direct, PubMed, Web of Science, Google Scholar and CNKI, covering publications from the inception of these platforms up to the year 2024. The search utilized the following keywords: glycocholic acid, synthesis processes, detection methods, and pharmacological effects. We have summarized the above content, prioritizing authoritative and valuable literature, and this review compiles a total of 134 relevant documents.

## 3 Physicochemical characteristics of glycocholic acid

Glycocholic acid is chemically designated as *N*-(3α,7α,12α-Trihydroxy-5β-cholan-24-oyl)-glycine. The chemical formula of the compound in question is C_26_H_43_NO_6_, with a molecular weight of 465.62. This compound exhibits weak acidity and appears as a white crystalline powder with a bitter taste. The compound exhibits limited solubility in water but is soluble in hot water and readily dissolves in organic solvents. In an aqueous environment, its degree of ionization is low, resulting in a pH value (water suspension) that ranging from 3.0 to 4.0. The melting point of glycocholic acid has been determined to be 130°C. However, it has been observed that the acid undergoes decomposition into glycine and cholic acid when exposed (Zhao et al., 2006).

## 4 Reactive formation of glycocholic acid

### 4.1 The glycocholic acid synthesis in the human body

**FIGURE 2:**
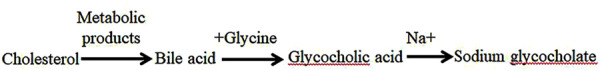
Body synthesis route map.

In the liver, cholesterol is subjected to intricate enzymatic processes that culminate in the generation of free bile acids. These bile acids are subsequently conjugated with glycine and taurine by the action of bile acid CoA synthase and amino acid N-acetyltransferase, forming conjugated bile acids such as glycocholic acid, taurocholic acid, taurodeoxycholic acid, and glycodeoxycholic acid (Jia et al., 2018; Ju et al., 2020). In physiological pH conditions, taurine conjugates are predominantly present in ionic form, whereas glycine conjugates exist in both ionic and molecular states simultaneously. In bile, the ratio of glycocholic acid to taurocholate is approximately 3:1 (Wu, 2013). The synthetic pathway of glycocholic acid within the body is shown in Figure 2.

### 4.2 Reactive synthesis of glycocholic acid

#### 4.2.1 Condensation agents method

**FIGURE 3:**
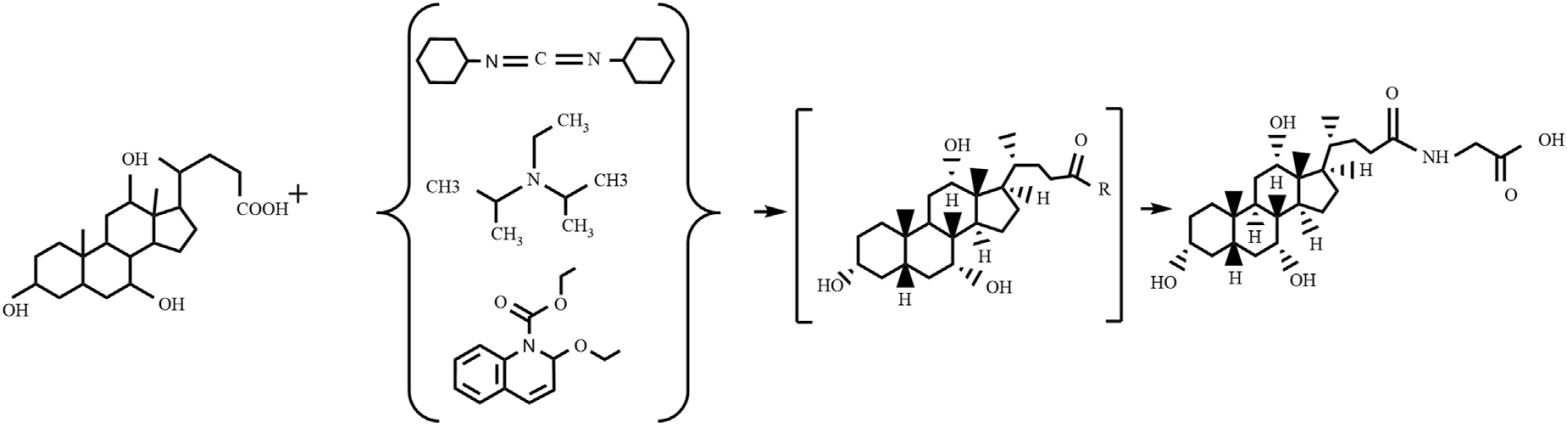
Preparation of glycocholic acid by the condensation agents method.

The condensation agents method (as shown in Figure 3) involves reacting specific condensing agents with bile acid to activate the carboxyl group, forming a reactive intermediate that can further react with the amine group of glycine or glycine methyl ester to form an amide bond, ultimately yielding glycocholic acid or its methyl ester (Ye et al., 2014). The most commonly used condensing agents are dicyclohexylcarbodiimide (DCC) (Cravotto et al., 2005), N,N-diisopropylethylamine (EDIA) (Ray et al., 2006), and 2-ethoxy-1-ethoxycarbonyl-1,2-dihydroquinoline (EEDQ) (Venturoni et al., 2012). This method has high production costs, and it is challenging to remove residual by-products, often requiring further purification processes, which is not conducive to large-scale use.

#### 4.2.2 Amide formation

Amide formation refers to the process in which a carboxylic acid reacts with an amine to produce an amide. This process can be carried out in various ways, including the use of coupling agents, inorganic bases, organic bases, or catalysts. Unlike the condensation agents method, amide formation is not limited to the use of specific coupling agents but covers a broader range of reaction conditions and catalysts. For instance, Momose et al. (1997) introduced a method for synthesizing glycocholic acid by simply mixing unconjugated bile acid, glycine ester, diethyl pyrocarbonate (DEPC), and triethylamine (Et_3_N) at room temperature for 30 min. This method is mild in conditions and can yield products with high purity, suitable for the relatively small-scale preparation of amide bile acid compounds.

#### 4.2.3 One-pot method

One-pot synthesis is a chemical strategy that allows all reactants to react directly in a single reaction vessel to complete the synthesis process. This method is favored for its simplicity and efficiency. In the synthesis of glycocholic acid, this approach is used to increase yield and simplify operational steps. Wang et al. (2021) used a one-pot method to synthesize glycocholic acid, starting with ursodeoxycholic acid and glycine ethyl ester hydrochloride as raw materials, and using N-carbamoyl chloride hydrochloride as a condensing agent under specific conditions. After the reaction was completed, hydrolysis was carried out in a sodium hydroxide aqueous solution, ultimately yielding glycocholic acid with a yield of over 90% (Wang et al., 2021). Additionally, Hitome et al. developed a new method for preparing 13C-labeled glycocholic acid, using 4-(4,6-dimethoxy-1,3,5-triazin-2-yl)-4-methylmorpholinium chloride (DMT-MM) as the condensing agent, which yields a higher yield than when using EEDQ as a condensing agent (Mitome et al., 2013). This method is simple and straightforward, employing a one-pot reaction to prepare relatively pure labeled glycocholic acid on a laboratory scale, suitable for clinical studies of breath tests.

#### 4.2.4 Mixed anhydride method

The mixed anhydride method (Gianluca et al., 2010) involves reacting cholic acid with ethyl chloroformate or methyl chloroacetate to synthesize a highly active mixed anhydride intermediate, which then reacts with glycine methyl ester. After purification, high-purity glycocholate acid methyl ester is obtained, and finally, an alkaline hydrolysis is used to obtain glycocholic acid or sodium glycocholate (SGC). This method is considered more appropriate due to its low raw material requirements and high utilization rate (up to 76%). Considering that ethyl chloroformate is a highly toxic substance, it can be replaced with the similar isobutyl chloroformate as an activating reagent to promote the formation of the amide bond (Cayley et al., 2007). For instance, Zhao et al. (2020) used the mixed acid anhydride method, utilizing triethylamine and isobutyl chloroacetate as catalysts, to synthesize glycocholic acid from bile acid and 4-aminobutyric acid. Although this method has high purity, the process is complex and incurs high costs.

#### 4.2.5 Hydrolysis

Hydrolysis is a chemical reaction process that can employ various catalysts and conditions, such as acidic, neutral, or alkaline environments, to accommodate different chemical structures and target products. For glycocholic acid (Ji et al., 2024), typically refer to the hydrolysis of glycocholic acid ethyl ester under alkaline conditions with sodium hydroxide or potassium hydroxide to break the ester bond and release glycocholic acid. Additionally, by consulting resources such as Chemical Book, it has been discovered that glycocholic acid can be obtained through an esterification reaction using cholic acid and glycine ethyl ester hydrochloride as raw materials, followed by a hydrolysis method.

#### 4.2.6 Other methods

The synthesis of glycocholic acid, in addition to the aforementioned methods, can also be carried out through the azide method (Zhu, 2019) and the acyl chloride method (Xu et al., 2010). The azide method encounters several challenges during the synthesis process, including high reagent costs, difficulty in clearing residues, and a substantial demand for raw materials. Moreover, the reagents used in the synthesis are sensitive to moisture, and the intermediates produced are toxic, posing potential health risks to operators and potentially harmful effects on the environment. Therefore, this method is not suitable for large-scale production. Similar to the azide method, the acyl chloride method also requires a large amount of raw materials in the synthesis of glycocholic acid, with a utilization rate is only 8.1%. Therefore, it is also not suitable for industrial large-scale production. Although these two methods have certain applications in the synthesis of glycocholic acid, their feasibility in industrial production is limited due to issues of cost and efficiency.

### 4.3 Extraction process of glycocholic acid

**FIGURE 4:**
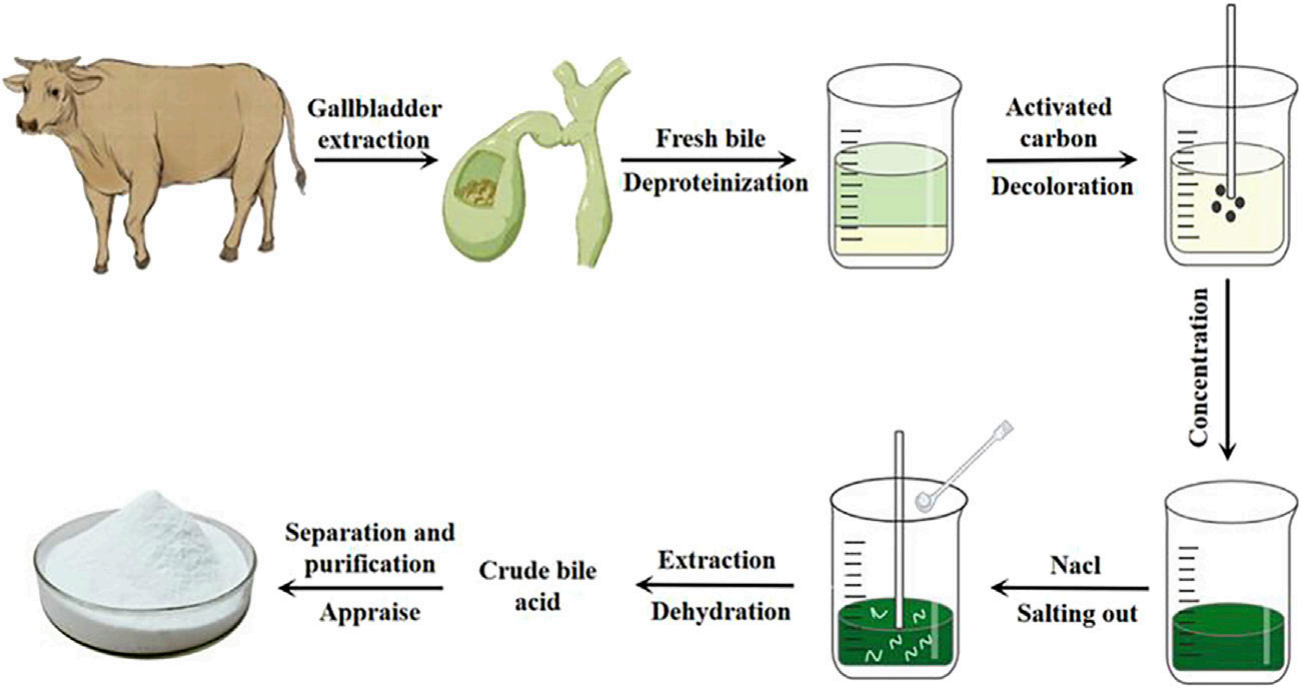
Extraction process of glycocholic acid.

Glycocholic acid is a conjugated bile acid that is typically mixed with various substances in biological matrices. To obtain high-purity glycocholic acid, a series of meticulous processing steps must be followed (Wang et al., 2020). The extraction and purification process is as follows (see flowchart in Figure 4).

#### 4.3.1 Raw material preparation and protein removal

Fresh bovine, ovine, or porcine bile is selected, filtered through gauze, and quantitatively collected (Chen and Luo, 2020). It is then mixed with 95% ethanol in a specific ratio. Most proteins and some pigments are removed through stirring, standing, and centrifugation, resulting in a green supernatant.

#### 4.3.2 Decolorization treatment

The supernatant is taken, and an appropriate amount of activated carbon is added. It is heated and stirred, then allowed to cool naturally before centrifugation to remove all pigments, yielding a light yellow supernatant.

#### 4.3.3 Ethanol recovery and salting out

Under specific conditions, all ethanol is recovered. A suitable amount of sodium chloride is added to the viscous bile and it is stirred until “white threads” appear, and then let it stand still.

#### 4.3.4 Extraction and dehydration

A mixture of chloroform and n-butanol is used for multiple extractions of the salted-out material. Anhydrous sodium sulfate is then added for dehydration, resulting in crude bile acids.

#### 4.3.5 Separation, purification, and identification

Column chromatography is employed, using suitable stationary phases and elution solvents based on the characteristics of glycocholic acid to separate it from other bile acids. The obtained samples are then identified, concentrated, and purified to yield white crystalline glycocholic acid.

## 5 Detection methods for glycocholic acid

### 5.1 Immunoassay

For bile acids with complex isomers, the detection and analysis present certain difficulties. Regarding the determination of bile acids, the main immunoassay methods include: homogeneous enzyme immunoassay (HEI), radioimmunoassay (RIA), enzyme-linked immunosorbent assay (ELISA), electrochemiluminescence (ECL), and latex enhanced immunoturbidimetry assay (LETIA). The principles and advantages and disadvantages of these methods are shown in Table 1.

**TABLE 1:** Immunoassay.

Principle	Name	Advantages and disadvantages	Limit of detection	Literature
By using the competitive binding analysis method, the labeled antigen and unlabeled antigen compete with the limited antibody for binding, forming the labeled antigen-antibody complex and the unlabeled complex. This allows the determination of the glycocholic acid content in the sample	HEI	The method is fast, sensitive, and easy to operate, but it is easily interfered by non-specific factors	0.3 μg/mL	Han and Chen (2019) and Huang (2019)
	RIA	Although this method has strong specificity and high sensitivity, there are issues such as isotope contamination during testing, inability to inactivate and degrade enzymes, and the occurrence of false positive reactions	0.1 μg/mL	Luo (1994)
	ELISA	The sensor’s sensitivity can reach the ng level, with a wide linear range, but it is only suitable for small-scale measurements	0.06 μg/mL	Zhang et al. (2016) and Cui et al. (2017)
	ECL	The technology of this method is complex and there is cross-contamination, so its application is not widespread	0.025 μg/mL	Zhang et al. (2015)
Based on latex micro particles sensitized with specific antibodies, the latex particles will agglutinate when they react with the corresponding antigen in the sample. The degree of latex agglutination is a function of the sample concentration, which can be used to quantitatively analyze the glycocholic acid content in the sample	LETIA	This method provides accurate, reproducible results with a wide linear range, but is susceptible to interference from cross-contamination. It is commonly used for emergency care testing	0.1 μg/mL	Liang et al. (2017)

**FIGURE 5:**
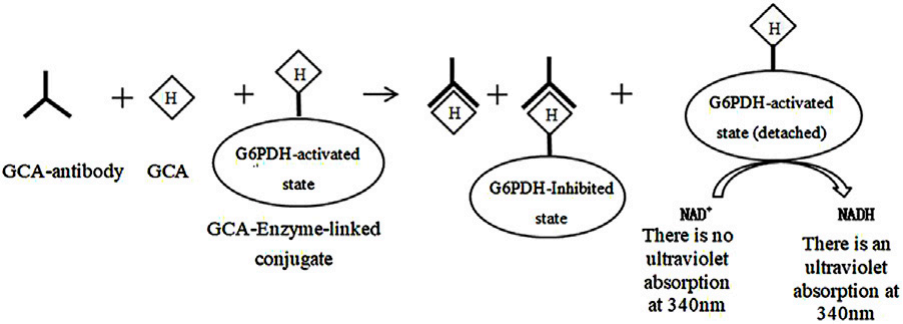
Principle of determination of glycocholic acid by homogeneous enzyme immunoassay.

The marker enzymes used in homogeneous enzyme immunoassays include digestive enzymes, malic dehydrogenase, β-D-galactosidase, and glucose-6-phosphate dehydrogenase, with glucose-6-phosphate dehydrogenase (G-6-PDH) being the most commonly used (Grote et al., 2012; Pandey et al., 2014; Roncancio et al., 2014). The glycocholic acid determination process is as follows (the route is shown in Figure 5): In brief, the glycocholic acid in the sample competitively binds to the anti-glycocholic acid specific antibody sites with the G-6-PDH-bile acid conjugate, which causes some of the bound enzyme-labeled drug to be released. It can be demonstrated that only the released enzyme-labeled bile acid conjugate can catalyze the conversion of NAD^+^ to NADH. Furthermore, the amount of NADH generated is proportional to the concentration of glycocholic acid in the sample. The NADH molecule exhibits a strong UV absorption at 340 nm; consequently, the change in absorbance observed before and after the conversion can be used to estimate the glycocholic acid content (Tao, 1987).

### 5.2 Chromatography

#### 5.2.1 Thin layer chromatography (TLC)

TLC is a relatively straightforward technique that yields results in a relatively short time. However, it can only qualitatively determine certain components by judging the depth of the fluorescent spots, which indicates the relative content of the components in the sample (Zhou et al., 2015). Yao et al. (2021) used the TLC method to identify the bile acid components in sheep bile, and the results showed that the bile acid components included CA, GCA, glycodeoxycholic acid (GDCA), GCDCA, TCA, taurodeoxycholic acid (TDCA), TCDCA and taurolithocholic acid (TLCA). The depth of the fluorescent spots indicated that the main bile acids in sheep bile were taurine-conjugated bile acids, with detection limits for each bile acid ranging from 4.0 to 10.4 ng (Yao et al., 2021).

#### 5.2.2 Liquid chromatography (LC)

##### 5.2.2.1 High performance liquid chromatography (HPLC)–ultraviolet detection

HPLC is a widely used detection method, apart from immunoassays. Its main characteristics are fast analysis speed and high separation efficiency. Li and Li (2009) established a HPLC method to detect the concentration of glycocholic acid in serum. They found that glycocholic acid showed a good linear relationship between the concentration range of 0.15625–10.0 μg/mL and the peak area. The lowest detectable concentration was 0.02 μg/mL, and the intra-day and inter-day RSD were both less than 5.00%, indicating that this method has high sensitivity, stability, and good separation effect for the determination of glycocholic acid (Li and Li, 2009).

##### 5.2.2.2 HPLC—evaporative light scattering detection (ELSD)

The bile acid components lack a conjugated system, which results in their weak absorption under ultraviolet conditions and heir susceptibility to interference by other substances. Therefore, the combination of evaporative light scattering technology can make up for the above problems, which is very suitable for the analysis of bile acid components (Zhang et al., 2009; Yao et al., 2021). For example, Shi et al. (2013) based on HPLC-ELSD technology, studied the bile acids and glycocholic acids in sheep gallbladder powder, and established a method that can simultaneously determine the contents of bile acids and glycocholic acids in sheep gallbladder powder (Yue et al., 2004). The method is straightforward to operate and exhibits a high degree of separation efficacy. The detection limits of this method can reach 0.49 μg/mL and can be used for the quality evaluation of sheep bile powder.

#### 5.2.3 Chromatography—tandem technique

##### 5.2.3.1 HPLC—tandem mass spectrometry (HPLC-MS/MS)

The combination of high performance liquid chromatography (HPLC) with mass spectrometry (MS) in HPLC-MS/MS technology allows for the effective separation of substances and the identification of their constituents with high accuracy and precision. This makes HPLC-MS/MS an increasingly popular choice for analytical identification (Wang, 2014). The HPLC-MS/MS method established by Mu et al. (2023) can complete the detection of six bile acids, including bile acid, cholic acid, taurocholic acid, glycocholic acid, taurodeoxycholic acid, and glycodeoxycholic acid, in plasma within 6 min, thus greatly reducing the detection time. The detection limits of this method range from 0.25 ng/mL to 0.45 ng/mL, while the quantification limits range from 0.84 ng/mL to 1.49 ng/mL (Mu et al., 2023). Bezoar is an animal medicine mainly used for detoxification, fever reduction, and tranquilization. However, the current phenomenon of adulteration has significantly compromised the quality of the medicinal material. Therefore, Liu et al. established a method to identify natural bezoar, artificial bezoar, and *in vitro* cultured bezoar, and found that the difference between the three types of bezoar is that a new compound, 3α,12α-dihydroxy-7-oxo-5α-cholic acid, was detected in the *in vitro* cultured bezoar, while glycocholic acid, glycodeoxycholic acid, and taurocholic acid were detected in both natural and artificial bezoar, however, the content of taurocholic acid was different, successfully distinguishing various types of bezoar medicinal materials (Liu et al., 2015).

##### 5.2.3.2 Gas chromatography (GC)-MS

GC-MS coupled technology combines the high-efficiency separation capability of gas chromatography for mixtures and the accurate identification capability of mass spectrometry for pure substances, and has been widely applied in various fields. Nevertheless, the inherent volatility of natural bile acid components is insufficient for GC analysis, so derivatization is needed to improve the volatility and thermal stability of the analytes (Wu and Han, 2020). Long et al. (2017) studied the differential metabolites in the serum of rats with acute liver failure using metabolomics, and analyzed the metabolic samples by GC-MS, which found 11 differential metabolites including glycocholic acid. This demonstrates that GC-MS can be used for the analysis of glycocholic acid.

## 6 The pharmacological effects of glycocholic acid

### 6.1 Anti-inflammatory effect

**FIGURE 6:**
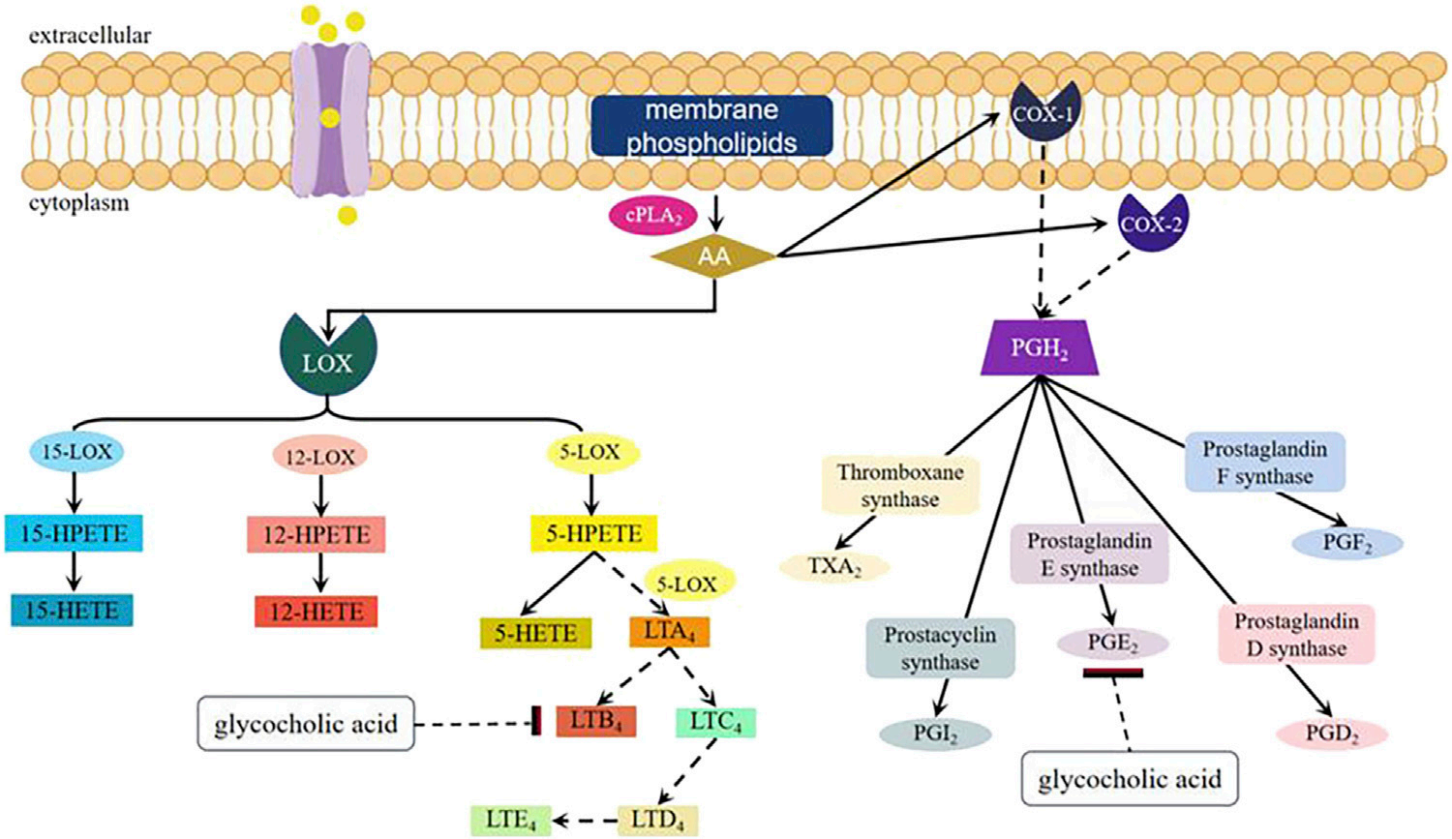
Metabolic pathway of AA and mechanism of action of glycocholic acid.

Arachidonic acid (AA) metabolites leukotriene B_4_ (LTB_4_) and prostaglandin E_2_ (PGE_2_) are the main inflammatory mediators released by cells, and play an important role in various acute and chronic inflammations. They can exhibit a strong inflammatory response even at extremely low concentrations, rendering them a valuable tool for investigating the underlying mechanisms of inflammation (Li et al., 2006). The experiment conducted by Li et al. (2006) has demonstrated that glycocholic acid can affect the cyclooxygenases (COX) and lipoxygenases (LOX) pathways in the AA metabolic pathway, thereby reducing the content of PGE_2_ and LTB_4_ in inflammatory tissues of rats, and has a significant inhibitory effect on acute and chronic inflammation (as shown in Figure 6). (Li, 2006) Nitric oxide (NO) is an important physiological mediator and intercellular and intracellular chemical messenger within the body. It is produced by nitric oxide synthase (NOS) from arginine and molecular nitrogen (Qiu et al., 2016). In the inflammatory response, inducible NOS (iNOS) is induced by inflammatory agents and mediators, leading to the synthesis and release of NO. Sustained high concentrations of NO can promote the occurrence of inflammation. Studies by Guan et al. showed that glycocholic acid significantly reduced the NO content in the peripheral blood of rats with Freund’s adjuvant-induced inflammation (44.48 ± 7.75 μmol·L^−1^) compared to the negative control group (155.93 ± 28.11 μmol·L^−1^), demonstrating that glycocholic acid has a significant inhibitory effect on the NO content in the peripheral blood of rats with Freund’s adjuvant-induced inflammation (Guan et al., 2009).

**FIGURE 7:**
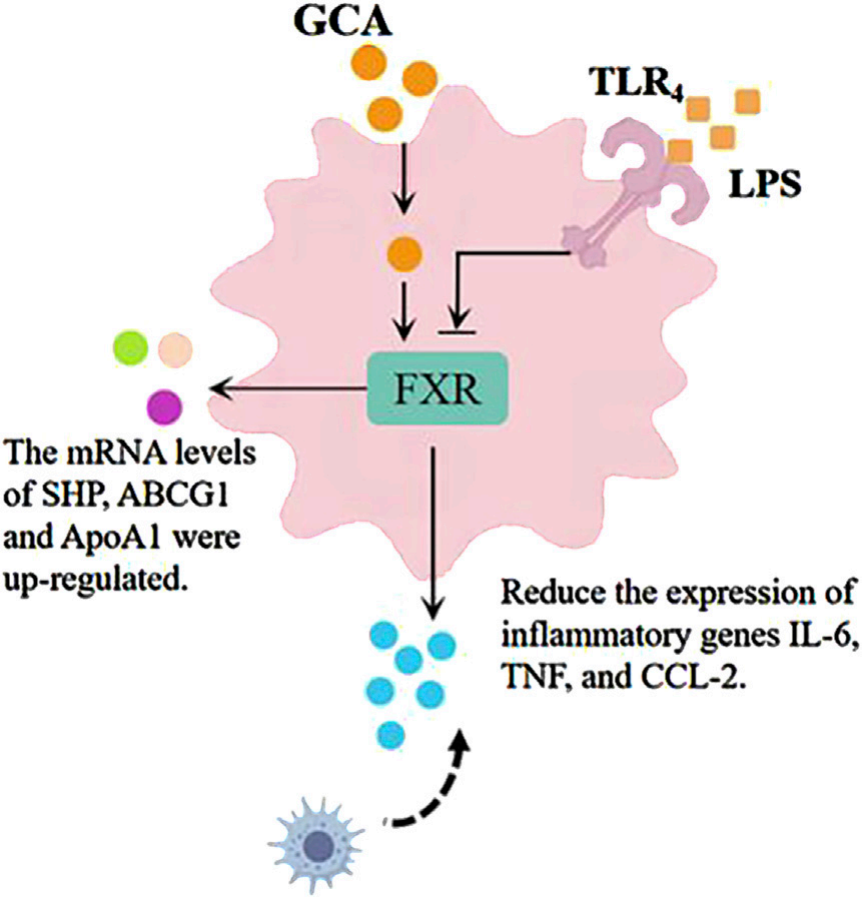
Anti-inflammatory mechanism diagram of glycocholic acid.

When inflammation occurs, the body rapidly synthesizes and releases various cytokines and chemokines, such as interleukin (IL) (Guo et al., 2023), interferon (IFN), tumor necrosis factor (TNF) (He et al., 2021), CXC chemokines, and CC chemokines, further enhancing the inflammatory response. Ge et al. (2023) found in a lipopolysaccharide-induced zebrafish inflammation model that tauroursodeoxycholic acid (TUDCA) could inhibit macrophage accumulation and suppress the upregulation of IL-6, TNF-α, and CCL-2 induced by lipopolysaccharide stimulation, reducing the transcriptional expression of these two cytokine (as shown in Figure 7). (Ge et al., 2023) The farnesoid X receptor (FXR) is a bile acid receptor that also plays an important role in the inhibition of inflammation (Wu et al., 2020a). The study indicates that glycocholic acid can increase the mRNA levels of downstream factors in the FXR signaling pathway, including the short heterodimer partner (SHP), ATP-binding cassette transporters G1 (ABCG1), and apolipoprotein A1 (ApoA1) (Ge et al., 2023). Additionally, glycocholic acid has an increasing trend in the expression of another bile acid receptor, the Takeda G protein-coupled receptor 5 (TGR5 or GPBAR1). In summary, glycocholic acid can inhibit the migration of macrophages and the cellular secretion of proinflammatory cytokines and chemokines, and its mechanism of action may be related to the upregulation of FXR expression.

### 6.2 Antioxidant action

**FIGURE 8:**
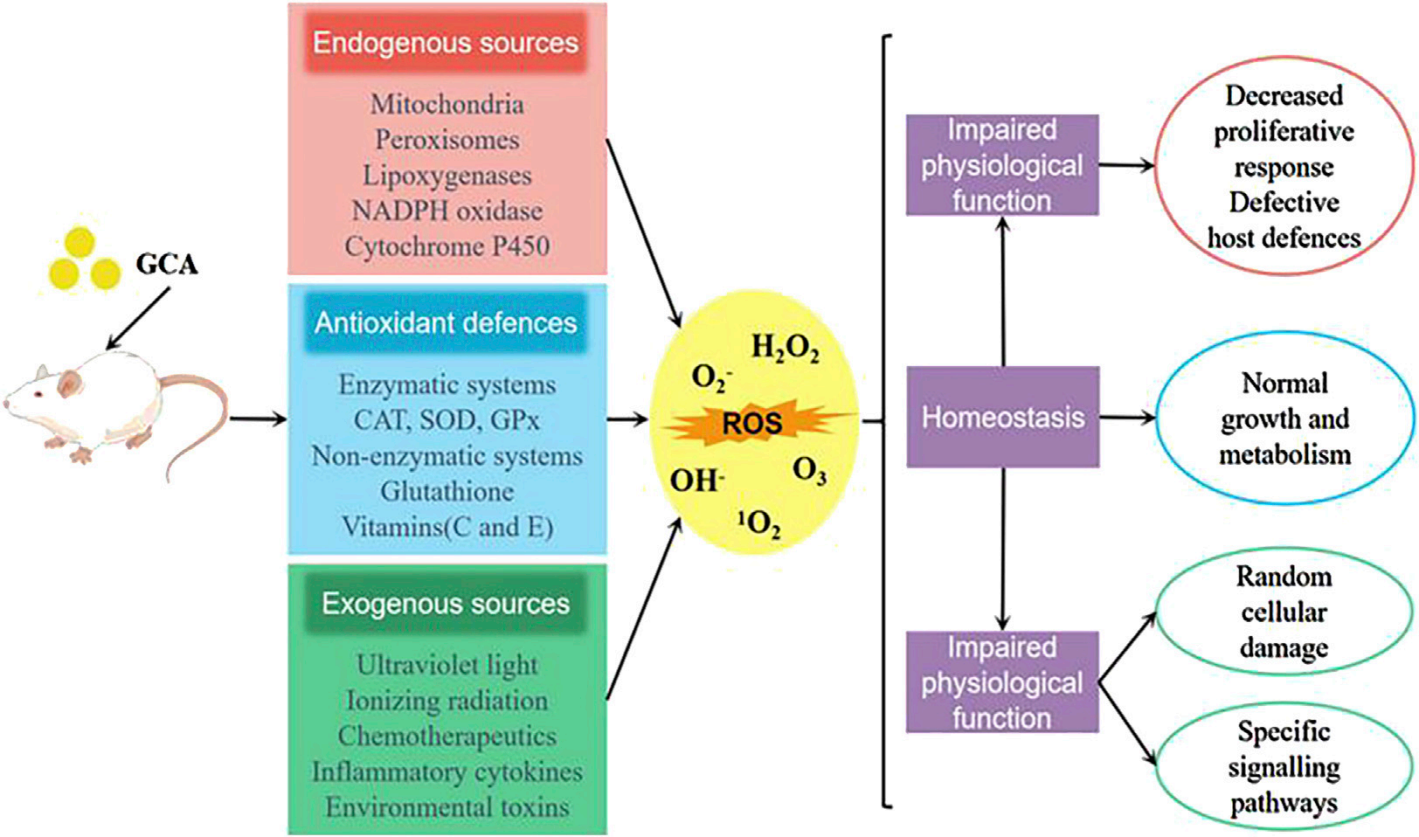
Sources of ROS and the antioxidant mechanism of glycocholic acid.

It is a well-established fact that biological organisms generate a large amount of reactive oxygen species (ROS) under the influence of the external environment and their own metabolism (Zhou et al., 2024). The overproduction of ROS not only impairs the normal signaling pathways of cells, but also oxidizes DNA, proteins, lipids, and causes tissue damage in the body (Zhang et al., 2019). Studies have shown (Wu et al., 2020b) that glycocholic acid significantly increase the activity of antioxidant enzymes such as glutathione peroxidase (GSH-Px) and superoxide dismutase (SOD) in mouse peritoneal macrophages. By reducing the content of malondialdehyde (MDA), glycocholic acid can reduce the oxidative damage of lipid peroxidation to mouse peritoneal macrophages (as shown in Figure 8), and regulate the antioxidant stress level of mouse peritoneal macrophages.

Age-related macular degeneration (AMD) is mainly characterized by a decrease in the ability of retinal pigment epithelial (RPE) cells to phagocytose and digest the outer segment discs of photoreceptor cells (Gong et al., 2021). This process results in the accumulation of undigested disc membrane remnants in the basal cytoplasm of RPE cells, which are then secreted and deposited on Bruch’s membrane, forming drusen. One of the treatment methods is to use antioxidants to prevent free radical damage to cells and protect photoreceptor cells. Warden and Brantley found that glycocholic acid can protect the tight junctions of RPE cells from extracellular oxidative stress, maintaining the localization of ZO-1 and transepithelial electrical resistance (TEER). *In vitro* experiments on cell migration and tube formation showed that glycocholic acid can inhibit vascular endothelial growth factor (VEGF)-induced choroidal endothelial cell (CEC) angiogenesis, indicating that glycocholic acid has a protective effect on AMD (Warden and Brantley, 2021).

### 6.3 Immunoregulatory function

**FIGURE 9:**
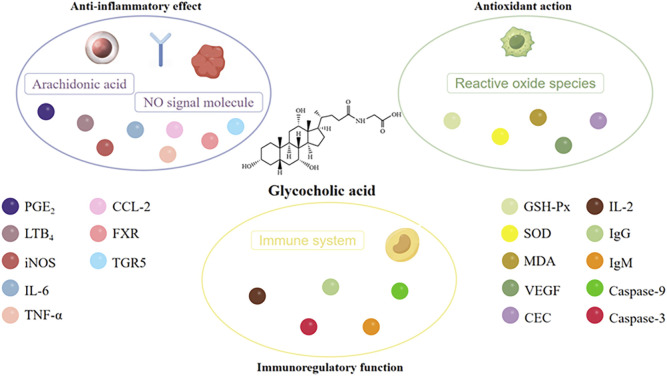
A molecular mechanism diagram of the pharmacological action of glycocholic acid.

The immune capacity of the body can be broadly divided into two categories: non-specific immunity and specific immunity. The former is primarily mediated by macrophages, while the latter is predominantly composed of immune organs and immune cells. The research results of Zhao indicated that the phagocytic index of the low-dose glycocholic acid was significantly higher than that of the control group and the CTX immunosuppression group (Zhao, 2006). It could significantly inhibit the delayed-type hypersensitivity (DTH) reaction in mice, while also increasing the IL-2 content in the serum, and significantly increasing the percentage of CD4^+^ cells in normal and CsA-inhibited mice (Li et al., 2007). The serum hemolysis method and ELISA method showed that the high and low dose glycocholic acid groups could significantly elevate the IgG and IgM content in the mouse serum. This evidence supports the conclusion that glycocholic acid has a regulatory effect on both non-specific immunity and specific immunity. Xin et al. (2011) studied the mechanism of glycocholic acid on the apoptosis of splenic lymphocytes, and found that glycocholic acid could induce nuclear condensation, apoptotic bodies and DNA fragmentation in splenic lymphocytes, significantly induce the externalization of phosphatidylserine in splenic lymphocytes, and increase the activity of Caspase-9 and Caspase-3. The higher the concentration of glycocholic acid, the more it can promote the apoptosis of splenic lymphocytes, indicating that glycocholic acid can balance the relationship between the body’s immunity and inflammation (Xin et al., 2011). The molecular mechanisms of glycocholic acid’s anti-inflammatory, antioxidant, and immunomodulatory effects are shown in Figure 9.

### 6.4 Antibacterial effect

Glycocholic acid belongs to the class of steroid compounds and is an amphiphilic biosurfactant with the ability to reduce surface tension. It works by altering the permeability of bacterial cell membranes, disrupting their integrity, and causing the leakage of intracellular substances, thereby inhibiting bacterial growth. Additionally, glycocholic acid may also affect the synthesis of bacterial proteins and nucleic acids, thereby inhibiting bacterial growth and reproduction. In recent years, it has been demonstrated that glycocholic acid has varying degrees of inhibitory effects on both Gram-positive and Gram-negative bacteria (Li, 2009). Dobson et al. (2018) isolated six bile acid derivatives, including glycocholic acid, from the broth culture of *Bacillus* amyloliquefaciens UWI-W23, and demonstrated that these six compounds had antimicrobial activity against *Pseudomonas aeruginosa* and *Bacillus* cereus. Furthermore, glycocholic acid also has an inhibitory effect on *Staphylococcus* (MICs = 15.6 μg/mL).

White *Candida* is a fungal pathogen with a high clinical antifungal drug resistance rate. It exists in small numbers in a normal host and does not cause disease. However, when the body’s immune function and general defense capacity decrease or the mutual restraint of the normal microbial flora is disrupted, it can proliferate extensively, change its growth form, and invade cells to cause disease. Kong et al. (2011) found that the antibacterial activity of glycocholic acid against white *Candida* is concentration-dependent. The antibacterial activity of glycocholic acid is weaker than that of cholic acid, possibly because the change in the C-24 side chain of the steroid nucleus alters the surface amphiphilicity, leading to a decrease in the affinity of glycocholic acid for the target of white *Candida* and its ability to transform the cell membrane.

### 6.5 Anti-breast disease action

Breast diseases are one of the major health hazards for women at present, mainly including mastitis, hyperplasia of mammary glands, fibroadenoma of the breast, intraductal papilloma, and breast cancer. Among these diseases, the incidence of breast cancer is high among female cancers, becoming one of the most common types of cancer (Huang, 2024; Zhang et al., 2024). Studies have shown that glycocholic acid may activate the FXR receptor and reduce the number of Treg cells in the tumor microenvironment by inhibiting the expression of the PI3K/AKT signaling pathway in Treg cells, thereby promoting tumor cell apoptosis and inhibiting tumor growth (Li et al., 2018; Ren et al., 2019; Xiang et al., 2019). Liang et al. (2021), in their research on the potential of glycocholic acid as a treatment for hyperplasia of mammary glands, established normal control group, model control group, tamoxifen group, and low, medium, and high dose groups of glycocholic acid. They found that after intervention with glycocholic acid, there was no significant enlargement of the rat’s breasts, and no obvious increase in breast diameter and nipple height. At the same time, the number of lobules and alveoli in the mammary glands decreased and began to atrophy, with a significant improvement in hyperplasia, indicating that glycocholic acid has an inhibitory effect on lobular hyperplasia and ductal dilation of the mammary glands (Liang et al., 2021). Further research has found that glycocholic acid has significant therapeutic effects in anti-tumor treatment, especially in the treatment of breast cancer. The experimental results prove that the tumor inhibition rate of glycocholic acid reaches 42.83%, and its specific target protein and RNA expression are significantly increased, effectively inhibiting the growth of mouse breast cancer cells. These findings provide strong scientific evidence for the development of glycocholic acid as a treatment drug for breast diseases (Liang et al., 2019).

## 7 Research on the application of glycocholic acid as an excipient

**FIGURE 10:**
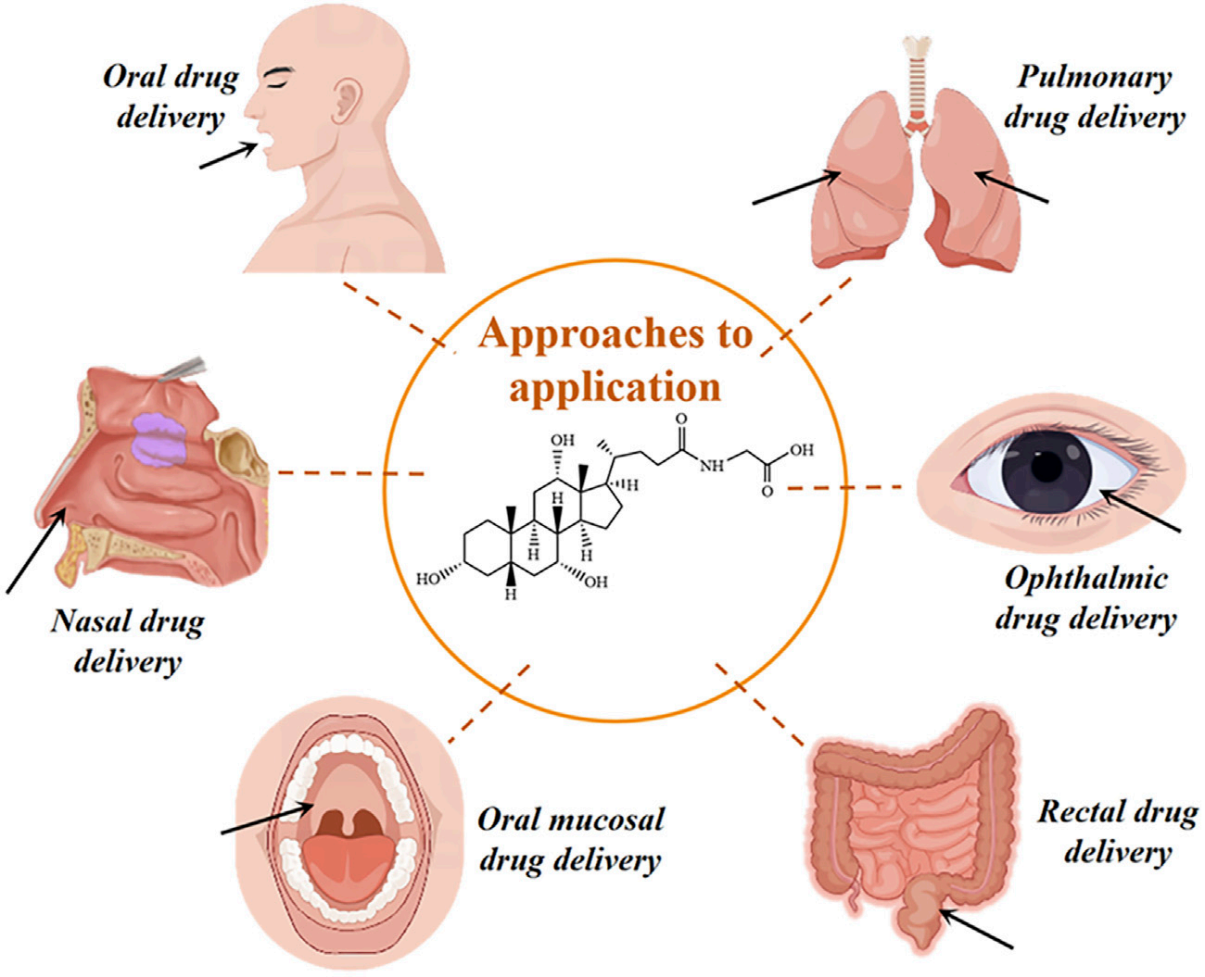
Application routes of glycocholic acid.

**FIGURE 11:**
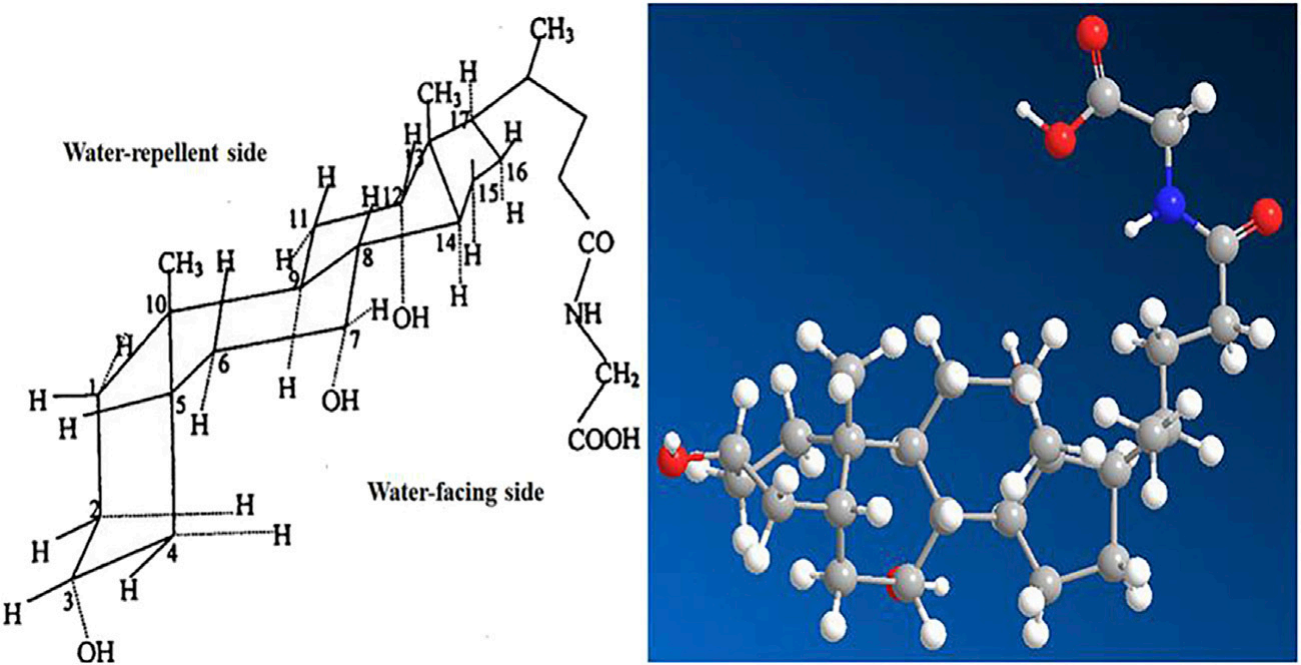
Stereostructure and ball-and-stick model of glycocholic acid.

Glycocholic acid is an endogenous substance present in the animal body, which is typically combined with sodium or potassium to form a salt. It is an amphiphilic biological surfactant with a planar rigid steroid skeleton, with its hydrophilicity coming from the hydroxyl group substituted on the concave (α) face of the steroid nucleus, and its hydrophobicity coming from the angular methyl group on the convex (β) face (Cai et al., 2020). This results in glycocholic acid exhibiting strong interfacial activity (Yang et al., 2021). Due to its special amphiphilic structure and good biocompatibility, it has been widely used as a drug carrier and absorption enhancer (Moghimipour et al., 2015), increasing the absorption of various drugs, mainly including oral, nasal, and oral administration routes (as shown in Figure 10). The stereo structure of glycocholic acid is shown in Figure 11 (Wang, 2016).

### 7.1 Oral drug delivery

Oral administration represents the most common method of drug delivery, offering a number of advantages. These include convenience, the avoidance of direct damage to the skin and mucous membranes, and the absence of pain caused by injection. However, the absorption of orally administered drugs is easily affected by factors such as gastrointestinal mucus layer, digestive enzymes, biological membrane permeability, intercellular tight junctions, and intrinsic solubility, so absorption enhancers are usually added to improve the bioavailability of drugs (Li et al., 2014; Huang and Sun, 2022; Song, 2022). As illustrated in Table 2, many researchers have studied the promotion of drug absorption by glycocholic acid through various pathways. R_1_ saponin, derived from Panax notoginseng, is a type of triterpenoid saponin isolated from Panax notoginseng, which has protective effects on the heart, nerves, and liver. However, studies have found that R1 saponin has poor water solubility and permeability, and is easily degraded in the gastrointestinal tract. Through the use of sodium glycocholate, the drug’s cellular diffusion is increased, resulting in a 3.25-fold increase in drug absorption in the duodenum.

**TABLE 2:** The absorption promotion effects of glycocholic acid and sodium salts on various drugs.

Route of administration	Type	Medicine	Animals/Model	The result	Literature
Oral	SGC- Liposomes (Lip)	Sanqi saponin R_1_	Rat, Caco-2 cells	Inducing cell membrane fluidity can significantly increase the absorption and bioavailability of water-soluble and low-permeability drugs	Fan et al. (2018)
	SGC-Lip, SGC	Insulin	Rat, Beagle, Caco-2 cells	The protease activity is significantly reduced, thereby facilitating increased insulin delivery to the absorption site, enhancing its glucose-lowering effect, promoting intestinal epithelial permeability, and improving bioavailability	Katsuma et al. (2006), Niu (2011), Niu et al. (2012), Hu et al. (2013), and Maroni et al. (2016)
	GCA- Mixed micelles, GCA- Lip	Vitamin K	Beagle, Caco-2 cells, Rat	Increase the contact area of the gastrointestinal tract, improve diffusion, disrupt the lipid arrangement of the cell membrane, enhance permeability to promote drug dissolution, and improve bioavailability (approximately 200%)	Sun et al. (2018) and Tong et al. (2018)
	GCA- micelle nanoparticles	Paclitaxel	Rat, Mouse	Due to the high affinity and intestinal targeting of GCA with the apical sodium-dependent bile acid transporter (ASBT), it significantly improves drug bioavailability, anti-tumor effects, and chemotherapeutic efficacy	Liu et al. (2023)
	GCA- micelle	Gemcitabine	Mouse, An *ex vivo* xenograft tumor model	The transport of the drug through the intestinal epithelial monolayer is mediated by the ASBT pathway. The pharmacokinetic data shows that the bioavailability of the modified micelle formulation is 81%, which is significantly higher than the unmodified micelle (20%), resulting in enhanced anti-cancer activity of the drug	Zang et al. (2023)
	SGC	Gentamicin, 5 (6)-Carboxyfluorescein	brush border vesicles、 Caco-2 cells	SGC leads to a decrease in TEER values, relaxing the tight junctions between cells, increasing paracellular permeability, and through the transcellular pathway, also increasing the permeability of Caco-2 cells, thereby enhancing drug absorption	Moghimipour et al. (2016)
	GCA-Lip	Exendin-4	Rat	The single oral dose (200 μg/kg) has a relative bioavailability of 19.5%, and the sustained pharmacokinetics lasts up to 72 h	Suzuki et al. (2019)
Nasal	SGC	Losartan	Calu-3 cells	Significant increase in mucosal permeability, enhancing drug diffusion. 1.0% SGC increases drug penetration by 7-fold, and this effect is concentration-dependent	Amoako-Tuffour et al. (2009)
		Insulin	Rabbit, Rat, Beagle	Reducing protease activity, increasing nasal mucosal permeability, forming temporary pores between epithelial cells, and significantly enhancing insulin absorption and efficacy	Harai et al. (1978), Aungst et al. (1988), Yamamoto et al. (1990), and Uchida et al. (1991)
		Enkephalin	Rat	Significantly increased drug absorption, rapid onset of action	Su et al. (1985) and Agu et al. (2004)
		Peptide T	Rabbit	Effective enhancement of nasal drug absorption, improved bioavailability, and the action is reversible within 4 h	Bagger et al. (2001)
Oral mucosal	SGC	Decitabine	Pig cheek mucosa	Analysis of the permeability-enhancing effects of four types of bile salts reveals that dihydroxy bile salts have a stronger permeability-enhancing effect compared to trihydroxy bile salts	Mahalingam et al. (2007)
		Mannitol	TR146 cells, Pig cheek mucosa	The permeability of drugs in two model systems is related to the concentration of bile salts, the degree of bile salt hydroxylation, and the type of interaction with bile salts	Nielsen and Rassing (1999)
		Acyclovir	Pig cheek mucosa	In the presence of SGC, drug permeability is increased 2–9 fold, and it is believed that the paracellular pathway is the main route for the oral transport of acyclovir	Shojaei et al. (1998)
		Fluconazole	Pig cheek mucosa	Under pH 5.8 conditions, 1.0% SGC increased the permeability (110-fold) and flux (75-fold) of flucytosine	Deneer et al. (2002)
		Antisense oligonucleotides (AONs)	Pig cheek mucosa	By effectively reducing the resistance of the cellular bypass, the oral permeability of the AON ISIS3082 compound is enhanced	Jasti et al. (2000)
		Propranolol hydrochloride	Pig cheek mucosa	At a certain concentration, SGC has a promoting effect on the permeability of drugs, and the permeability coefficient varies with changes in the pH and osmotic concentration of the medium, but is not affected by the drug concentration	Xu and Fang (2001)
Rectal	SGC	Insulin	Rabbit	Reduce the barrier function of the rectal membrane, increase the proportion of drug monomers, protect the drug from being hydrolyzed by proteases, and enhance the permeation effect	Yamamoto et al. (1992)
		Amoxicillin sodium	Rat	The average Cmax of the drug increased with the increase of SGC concentration, and the average Cmax and average AUC were 24.5 and 25 times higher, respectively, compared to the group using the drug alone	Mishima et al. (1995)
Other	SGC	Insulin	Rabbit	Increasing drug absorption: Within a certain range, the absorption effect increases with the increase in concentration. At higher pH values, ocular absorption is more effective	Yamamoto et al. (1989) and Xuan et al. (2005)
		Peptides Non-peptide (Atenolol, Timolol)	Rabbit	By weakening the tight junction barrier of the epithelial cells and improving the mucus layer, the conjunctival permeability of peptide and non-peptide drugs can be enhanced to varying degrees	Hayakawa et al. (1992)
		Insulin	Rat	After adding absorption promoters, the drug’s solubility in the lungs is increased, the degradation rate is slowed down, and the hypoglycemic effect is sustained and significant	Yamamoto et al. (1994), Li et al. (2000a), and Li et al. (2000b)

### 7.2 Nasal drug delivery

For small doses of drugs that are unstable in the gastrointestinal tract, nasal administration is a suitable route of administration. It has become a research focus due to its advantages of rapid onset, convenient administration, and avoidance of first-pass effect of the liver (Liao et al., 2023). However, there are two main limiting factors for nasal absorption: the low membrane permeability of the drug and the ciliary clearance effect of the nasal mucosa (Zhang et al., 2022). To overcome these issues, methods such as adding absorption enhancers can be used to improve drug absorption (as shown in Table 2), such as peptide substances like insulin, which are easily degraded by proteases and have poor permeability. The above method can significantly increase the drug activity and enhance the therapeutic effect. The mechanisms of action may include the following (Donovan et al., 1990; Shao and Mitra, 1992; Lin et al., 2007): 1) affecting the thermodynamic properties of the drug in solution; 2) changing the molecular structure of cell membranes by forming membrane pores; 3) loosening the tight connections between epithelial cells; 4) inhibiting protease activity in the mucosa; 5) altering the mucus properties of the respiratory tract to reduce diffusion barriers.

### 7.3 Oral mucosal drug delivery

Oral mucosal drug delivery is becoming increasingly being recognized for its ease of use and removal, avoidance of degradation by gastrointestinal enzymes, direct entry into the systemic circulation after absorption through mucosal tissue, and avoidance of the first-pass effect. The factors that influence the delivery of drugs through the oral mucosa can be divided into two main categories: those that affect the permeability of the mucosa and those that affect the viscosity of the mucus and saliva. Absorption enhancers can reversibly alter the stratum corneum barrier of the oral mucosa to promote transmucosal drug absorption, and they have been widely used in oral drug permeation studies (as shown in Table 2). (Li and Huang, 2012; Liu et al., 2014; Chen et al., 2023) Decitabine is a natural adenosine analogue of 2′-deoxycytidine, and it is currently the most potent DNA methyltransferase inhibitor. However, decitabine has poor chemical stability and is not suitable for intravenous injection. Therefore, researchers have evaluated the feasibility of oral administration of decitabine and the influence of four bile salts (sodium taurocholate, STC; SGC; sodium deoxytaurocholate, SDTC; and sodium deoxyglycocholate, SDGC) on its permeability. It was found that dihydroxy bile salts (SDTC, SDGC) had a stronger enhancing effect on the permeation of decitabine through porcine buccal mucosa. The flux was found to be increased by 28–43 times when trihydroxy bile salts were present at a concentration higher than 10 times the critical micelle concentration (CMC), while dihydroxy bile salts only needed a concentration higher than 3 times the CMC to achieve the same flux.

### 7.4 Rectal drug delivery

Rectal administration refers to the method of delivering drugs into the intestine through the anus, where they are rapidly absorbed through the rectal mucosa into the systemic circulation, exerting their pharmacological effects to treat systemic or local diseases (Zhou et al., 2022). Drug absorption in the rectum is generally through passive diffusion, either directly through epithelial cells (transcellular) or through tight junctions (paracellular) across the rectal wall (Li et al., 2019). Glycocholic acid can bind with sodium and calcium ions, altering the tight junctions of cell membranes, leading to a decrease in the TEER value of the Caco-2 cell monolayer model, increasing the transport of hydrophilic macromolecules through the paracellular route, and inhibiting the activity of drug-metabolizing enzymes at the site of drug action (Lindhardt and Bechgaard, 2003).

### 7.5 Other administration methods

The majority of peptides and proteins are ineffective when taken orally, and injection administered is hindered by the degradation of proteases and the barrier of the mucosa. Consequently, alternative routes of administration are highly desirable (as shown in Table 2), such as ocular administration and pulmonary inhalation. The alveolar epithelium, which is characterised by a large surface area, facilitates the absorption of numerous drugs that are poorly absorbed in the intestine and other sites due to the short pathway to the bloodstream.

## 8 The significance of serum glycocholic acid testing

In normal physiological conditions, the liver, as the main metabolic organ in the human body, is able to effectively reabsorb glycocholic acid, resulting in a very little glycocholic acid entering the blood circulation, usually less than 1%. It can be concluded that the concentration of glycocholic acid in the peripheral blood is extremely low, with fasting serum glycocholic acid concentration less than 2.50 mg/L, which can be used to assess the health status of the hepatobiliary system (Yao et al., 2003; Liu, 2012). However, when the liver is injured or there is bile duct obstruction, the liver’s absorption and secretion of glycocholic acid becomes abnormal, resulting in an increase in serum glycocholic acid content. Therefore, serum glycocholic acid can be used as a sensitive indicator to evaluate liver cell function and the metabolic function of the hepatobiliary system. Studies have shown that the serum concentration of glycocholic acid is elevated to varying degrees in pathological conditions such as acute hepatitis, chronic hepatitis, cirrhosis, and liver cancer (Zhang et al., 2017). Kong et al. (2011) conducted a comparative analysis of serum glycocholic acid and total bile acid levels between individuals with different liver and biliary diseases and a control group (Kang et al., 2018). The serum glycocholic acid levels were 152.8 ± 78.2 μg/dL in the control group, 205.8 ± 72.4 μg/dL in hepatitis B virus carriers, 2,827.8 ± 945.7 μg/dL in acute hepatitis, 2,292.5 ± 712.3 μg/dL in chronic hepatitis, 3,592.8 ± 1,548.9 μg/dL in cirrhosis, and 3,992.2 ± 1,648.2 μg/dL in liver cancer, showing varying degrees of elevation.

Furthermore, serum glycocholic acid has certain reference value for the diagnosis and prognosis of acute and chronic hepatitis, can assess the activity of chronic hepatitis, predict the prognosis of cirrhosis, and also indicate other digestive diseases (Xia, 1995; Bao and Bao, 1998). Liu demonstrated that in patients presenting with acute icteric hepatitis during the jaundice period, the positive rates of serum alanine aminotransferase and serum glycocholic acid were both 100% (Liu, 2012). However, during the recovery period, although the routine liver function indicators had returned to normal, the serum alanine aminotransferase conversion rate was 97.87%, while the serum glycocholic acid was only 46.67%, indicating a potential risk of developing into chronic hepatitis. Therefore, using serum glycocholic acid as a discharge criterion is necessary (Zhang and Zhu, 1997).

## 9 Clinical researches on glycocholic acid

Congenital bile acid synthesis disorders are caused by enzyme deficiencies during the synthesis process and typically manifest in newborns or infants, though they can also present in adulthood. Symptoms include cholestasis, fat-soluble vitamin deficiencies, coagulopathy, chronic liver disease, growth retardation, or neurological impairment. Studies have shown that glycocholic acid can treat bile acid synthesis disorders due to bile acid-CoA: amino acid N-acyl transferase (BAAT) deficiency (Setchell et al., 2013). In 2012, the Cincinnati Children’s Hospital Medical Center in the United States advanced to Phase III clinical trials for the use of glycocholic acid in treating patients with congenital bile acid synthesis disorders. Over a span of 10 years, they selected male and female patients aged between 1 week and 85 years. The primary measurement was the change in atypical bile acid synthesis in urine, analyzed by mass spectrometry, while secondary measurements included liver function tests, fat-soluble vitamin absorption, growth status, and the incidence and severity of adverse events. The results indicated that after oral administration of 15 mg/kg glycocholic acid, the total duodenal bile acid concentration was 23.3 ± 19.1 mmol/L, with 63.5% ± 4.0% of bile acids secreted in the conjugated form, of which glycocholic acid represented 59.6% ± 9.3% of the total bile acids, demonstrating effective intestinal absorption of glycocholic acid (Heubi et al., 2015). Additionally, the absorption of vitamin D2 and tocopherol significantly improved after oral administration of glycocholic acid. Growth status also showed marked improvement in prepubertal patients with growth retardation, and no adverse reactions were reported. Therefore, we speculate that glycocholic acid holds very promising prospects for treating bile acid amidation defects and may become an approved medication in the future.

## 10 Conclusion and perspectives

In summary, this article provides a comprehensive review of the synthesis and extraction techniques, detection methods, pharmacological effects, and clinical applications of glycocholic acid. The synthesis pathways of glycocholic acid include both *in vivo* synthesis and chemical synthesis. The synthesis of glycocholic acid in the body primarily relies on the conversion of cholesterol in the liver through the classical pathway and the alternative pathway, which involves a series of enzymatic reactions to generate primary bile acids. These primary bile acids then combine with glycine to form glycocholic acid or its salts. The produced glycocholic acid salts can enter the gallbladder through the action of the bile salt export pump (BSEP), multidrug resistance-associated protein 2 (MRP2), and multidrug-resistant protein 3 (MDR3), and are secreted into the small intestine with bile to participate in the digestion, absorption, and transport of lipids and fat-soluble vitamins. Most of the glycocholic acid salts are reabsorbed at the terminal ileum of the small intestine through the apical sodium-dependent bile acid transporter (ASBT), entering the portal vein circulation and returning to the liver to complete the enterohepatic circulation process. In the field of chemical synthesis, the preparation of glycocholic acid primarily employs the following methods: condensation agents method, mixed anhydride method, one-pot method, and amidation reaction. The core mechanism of these methods involves the reaction between the carboxyl group and the amine, forming an amide bond to synthesize the target compound. Among them, the condensation agents method, although operationally simple, is highly dependent on the condensation agents used, and the reaction tends to leave by-products, requiring additional purification steps for removal. The mixed anhydride method, while using fewer raw materials and yielding high-purity products, requires strict reaction conditions, and the instability of the mixed anhydride intermediates also increases the complexity of the operation. The one-pot method features simplified operational steps and atomic economy, making it particularly suitable for target molecules that require multi-step synthesis. The amidation reaction can use different condensation agents and conditions, suitable for a wide range of combinations of carboxylic acids and amines, and by changing different combinations, a variety of amide compounds with diverse structures can be obtained. Overall, each method has its own advantages and limitations. The choice of method depends on the structure of the target compound and the required reaction conditions, as well as specific requirements for yield, purity, and operational convenience. In practical applications, researchers need to flexibly choose or optimize the most appropriate synthetic strategy based on the experimental purpose and conditions.

In terms of detection technology, immunoassays and chromatography are currently the mainstream methods for detecting glycocholic acid. In clinical practice, RIA is widely used to analyze the levels of glycocholic acid in serum, which is of great significance for assessing the severity of liver disease, monitoring disease progression, and predicting prognosis. *In vitro* detection methods include TLC and LC; the former is mainly used for qualitative analysis, while the latter can, based on the structural characteristics of glycocholic acid, select appropriate detectors and hyphenated techniques to achieve quantitative analysis of glycocholic acid samples.

As research continues to delve deeper, the various pharmacological activities of glycocholic acid are gradually being revealed, including anti-inflammatory and antioxidant effects. Activation of FXR reduces the synthesis of liver lipoproteins, as well as the levels of plasma triglycerides and cholesterol. This is because FXR activation induces the expression of genes involved in lipoprotein metabolism or clearance, while simultaneously suppressing the expression of genes involved in triglyceride synthesis. Furthermore, the activation of FXR can also inhibit the expression of transcription factor SREBP-1c and its downstream hepatic lipid synthesis genes by inducing the expression of SHP, thereby reducing hepatic lipid synthesis. Therefore, it is inferred that glycocholic acid may play a role in regulating glucose and lipid metabolism and inhibiting the expression of gluconeogenesis-related genes. Due to its unique structure, glycocholic acid can improve the oral bioavailability of drugs with low water solubility and low permeability through the formation of micelles and saponification. As a pharmaceutical excipient, glycocholic acid can form mixed micelles with phospholipids, serving as a vehicle for the injection administration of insoluble drugs. This characteristic gives it potential application value in drug delivery systems, especially in improving drug solubility and bioavailability. In summary, the pharmacological activities and drug delivery potential of glycocholic acid make it a compound worthy of further research. With the growth of the modern pharmaceutical field, we can anticipate that glycocholic acid and its derivatives will play an increasingly important role in drug development and the treatment of various diseases.

Through a comprehensive analysis of domestic and international data, we have found that research on glycocholic acid is still in a relatively early stage, and there are several main issues: Firstly, the synthesis process is relatively outdated and needs further optimization to improve efficiency and reduce costs. Secondly, the application range of detection technology is limited, and more hyphenated techniques need to be developed to enhance the accuracy and sensitivity of analysis. Thirdly, research on the physiological activities and pharmacological mechanisms of glycocholic acid is not deep enough, and further exploration of its specific mechanisms of action in organisms is required. Lastly, there have been no reports on the development of glycocholic acid into formulations, indicating that there is still a lot of room for development in clinical applications and drug development. Therefore, future research on glycocholic acid compounds should focus on the following areas: Firstly, optimizing the synthesis process to improve synthesis efficiency and product purity; secondly, developing and applying new detection technologies, especially hyphenated techniques, to enhance the accuracy and sensitivity of analysis; thirdly, conducting in-depth studies on the physiological activities and pharmacological mechanisms of glycocholic acid to provide a scientific basis for clinical applications. At the same time, strengthening the research on the combination of glycocholic acid drugs with other medications may better exert therapeutic effects, meet the medication needs of a broad range of patients, and promote the development of traditional Chinese medicine.
